# Obesity surgery and satiety control

**DOI:** 10.1590/S1516-31802006000400001

**Published:** 2006-05-04

**Authors:** José Ernesto dos Santos

Obesity, i.e. body mass index (BMI) above 30 kg/m^2^, is a sign and symptom that has variable etiology, clinical picture and therapeutic response. In Brazil, it became a public health problem some time ago. There are few doubts that lifestyle changes provoked or imposed by “acculturation” processes are related to the increasing prevalence of obesity.

The failure of traditional clinical treatment is particularly marked in morbidly or severely obese subjects (BMI > 40 kg/m^2^), who represent 1% to 2% of all obese patients and whose prevalence has been significantly increasing in some localities. In Ribeirão Preto, for example, the prevalence of severely obese individuals in the adult population was 0.8% in 1984 and increased to 2.2 by 2002.* These patients have high prevalence of clinical complications and high mortality.^1^ Clinical treatment by classical dietary techniques is frustrating and no anorexigenic medications that could safely be used on a long-term basis are yet available.

Studies conducted on populations within different cultures have demonstrated that these patients ingest food when stimulated by various situations other than hunger, and that they have difficulty in perceiving satiety.^2^ Anxiety, anguish and other signs and symptoms may be associated with increased food ingestion.^3^

Over the last few years, surgical treatment of morbid obesity has been gaining popularity. Its efficacy in provoking weight loss has been confirmed by well controlled studies conducted especially in the United States^4,5^ and Sweden.^6^ Surgical treatment causes a marked weight loss, which is maximal at about one year after surgery. In some reports, this weight loss has been found to substantially improve the patients’ quality of life.^7^

A paper by Santoro et al.^8^ on this topic appears in this issue of the São Paulo Medical Journal. The authors describe the rationale, technique and preliminary results of a new surgical procedure for the initial treatment of morbidly or severely obese patients. They justify the new technique by presenting a good review of the physiological aspects of hunger and satiety control and, on the basis of the evidence reviewed, they propose a surgical procedure consisting of stomach reduction, omen-tectomy and partial enterectomy, leaving about three meters of small intestine. The rationale is based on the fact that this surgical procedure creates a “new digestive tract” adapted to the sociocultural eating conditions of the 21^st^ century.

The authors applied the technique to a significant number of patients (52 women and 48 men), with clinical and surgical follow-up of one to 29 months. The results, which are promising, are evaluated in terms of weight loss, appearance of the classical complications of major gastrointestinal surgery, and the patients’ subjective evaluation of satiety.

A series of problems must be analyzed when proposing the use of existing surgical techniques for the treatment of severely obese patients. The Journal of the American Medical Association (JAMA)^9^ recently published a retrospective study of more than 16,000 operations performed in the United States. Surprisingly, this showed a much higher mortality rate than reported previously. Mortality at 30 days, 90 days and one year after the surgery was 37%, 4.8% and 7.5% for men and 1.5%, 2.1% and 3.7% for women, respectively. In addition, mortality was significantly higher for older patients, reaching 11.1% after one year for obese patients older than 65 years. Furthermore, in the same issue of JAMA, Zingmond et al.^10^ analyzed the frequency of hospitalization of a group of patients before and after surgery for weight reduction. These authors observed that hospitalization increased within a ten-year period (19.3% *versus* 7.9%). Another matter that deserves special attention is that medium and long-term reduction of ingestion and malabsorption (variable, but always present during the postoperative period) may lead to nutritional deficits that sometimes are difficult to diagnose. Anemia, osteoporosis and other vitamin and mineral deficiencies have been described.^11^ There is a need for special supplements during the postoperative period, but in quantities and for periods of time that are still undetermined.

In short, the article by Santoro et al.^8^ makes an important contribution to the search for an efficient and safe surgical technique for treating severely obese patients. However, care should be taken to ensure that such surgical procedures are only performed in specialized centers, on patients selected according to clinical and psychological criteria and by experienced surgeons with backup from multiprofessional teams for long-term follow-up.

## Footnotes

*
*Dos Santos JE, Dressler WW. Variação de fatores de risco para doença arterial coronariana em 10 anos em Ribeirão Preto. In preparation.*
